# Increased Risk of Major Depressive Disorder Following Tinnitus: A Population-Based Study

**DOI:** 10.3389/fneur.2022.836842

**Published:** 2022-03-21

**Authors:** Herng-Ching Lin, Sudha Xirasagar, Chia-Hui Wang, Yen-Fu Cheng, Tzong-Hann Yang

**Affiliations:** ^1^Sleep Research Center, Taipei Medical University Hospital, Taipei, Taiwan; ^2^School of Health Care Administration, College of Management, Taipei Medical University, Taipei, Taiwan; ^3^Department of Health Services Policy and Management, Arnold School of Public Health, University of South Carolina, Columbia, SC, United States; ^4^Department of Urban Development, University of Taipei, Taipei, Taiwan; ^5^Research Center of Sleep Medicine, College of Medicine, Taipei Medical University, Taipei, Taiwan; ^6^Department of Medical Research, Taipei Veterans General Hospital, Taipei, Taiwan; ^7^Department of Otolaryngology-Head and Neck Surgery, Taipei Veterans General Hospital, Taipei, Taiwan; ^8^Faculty of Medicine, National Yang Ming Chiao Tung University, Taipei, Taiwan; ^9^Institute of Brain Science, National Yang Ming Chiao Tung University, Taipei, Taiwan; ^10^Department of Otorhinolaryngology, Taipei City Hospital, Taipei, Taiwan; ^11^Department of Speech, Language and Audiology, National Taipei University of Nursing and Health, Taipei, Taiwan; ^12^University of Taipei, Taipei, Taiwan

**Keywords:** tinnitus, depression, epidemiology, otology, neuro-otology

## Abstract

**Background and Purpose:**

In this study, we aimed to evaluate the relationship between tinnitus and a subsequent diagnosis of major depressive disorder (MDD) by studying the incidence of both entities.

**Design:**

A retrospective cohort study.

**Methods:**

Data for this observational follow-up study were retrieved from the Taiwan's National Health Insurance Dataset. A total of 375,272 patients with newly diagnosed tinnitus (study group) were retrieved. The date of first diagnosis of tinnitus was assigned as their index date. Comparison patients were selected by propensity score matching (one per case, *n* = 375,272 controls) from the same dataset, with their index date being the date of their first health service claim in the year of diagnosis of their matched index case. We tracked each patient's claims records for 1 year from the index date to identify those who received a diagnosis of MDD. Cox proportional hazards regression was performed to calculate the MDD hazard ratio for cases vs. controls.

**Results:**

We found that the overall incidence rate for MDD was 0.78 (95% CI = 0.76~0.80) per 100 person-years, being 1.17 (95% CI = 1.14~1.21) among the study cohorts and 0.38 (95% CI = 0.36~0.40) among the comparison cohorts. The log-rank test revealed that the patients in the study cohort had significantly lower one-year MDD-free survival when compared to the comparison cohort (*p* < 0.001). Cox proportional hazards analysis showed that the patients in the study cohort had a higher hazard of developing MDD than the patients in the comparison cohort (adjusted HR = 3.08, 95% CI = 2.90~3.27).

**Conclusions:**

In this study, we demonstrate that tinnitus is associated with an increased hazard of subsequent MDD in Taiwan.

## Introduction

Tinnitus is defined as a non-observable, self-reported auditory phantom phenomenon with patients internally perceiving a sound without a corresponding external sound source (1, 2). Tinnitus affects ~50 million adults in the United States, the prevalence ranging from 4.6 to 32.9 % in the world (3–5). Subjective loudness, quality, and awareness of tinnitus sounds varies widely, leading to varied degrees of annoyance, ranging from slight annoyance to severe distress (6–8). In 20% of the cases, tinnitus causes distress that manifests as annoyance, anxiety, insomnia, problems of concentration, and depression (9).

Major depressive disorder (MDD) is a psychiatric illness with feelings of sadness, guilt, hopelessness, and worthlessness, followed by a high probability of suicide (10). It is recurrent, disabling, and widely prevalent with estimated lifetime prevalence of 20%. It is also a leading cause of disability and mortality in the world (11). Although recognized as the leading cause of disease burden worldwide, the actual pathogenesis of MDD remains unknown (10, 11).

One study proposed the term “tinnitus disorder” defined as tinnitus with associated sufferings, including emotional distress, cognitive dysfunction, and/or autonomic arousal, leading to behavioral changes and functional disability (12). In clinical practice, it is often difficult to identify whether higher tinnitus distress leads to a depressive mood or *vice versa*. Tinnitus and MDD may share common symptoms and risk factors, such as chronic health problems, lower socioeconomic status, and higher perceived stress levels (13). MDD is also potentially related to tinnitus due to the similarity of pathophysiology (14, 15). The literature shows findings that tinnitus sufferers often have a comorbid depression; however, a causal relationship and direction of causation remain uncertain because of cross-sectional study designs and use of self-reported data from surveys in these studies (13, 16–19). Recently, some investigators have reported that tinnitus may be a trigger factor in depression, whereas others have indicated that the association between depression and tinnitus may be bidirectional, rather than unidirectional (14, 16). Nonetheless, to our knowledge, no population-based studies have investigated the association between tinnitus and subsequent MDD.

This study aimed to investigate the relationship between tinnitus and MDD using a large, population-based dataset in Taiwan by studying the incidence of both entities.

## Methods

### Database

We obtained data on the study population from Taiwan's National Health Insurance Research Database (NHIRD), which is maintained and regulated by the Data Science Center of the Ministry of Health and Welfare of Taiwan. The NHIRD was established in 1995 and covers more than 99.6% of all 23 million-plus citizens. The NHIRD includes data on registries of contracted medical facilities and beneficiaries, and claims submitted for reimbursement. Taiwan's NHI program covers virtually everything, including preventive care, cancer screenings, mental health, general primary care, and in-hospital care. The NHIRD provides a unique opportunity for scientists in Taiwan to investigate the association between tinnitus and the subsequent occurrence of major depressive disorder. The study was approved by the Institutional Review Board of Taipei Medical University (TMU-JIRB N202109059). Since we used an administrative claim dataset to analyze data, patient informed consent was waived.

### Study Sample

We designed this study as an observational cohort study. We established the study cohort by identifying 393,761 patients aged ≥ 20 years who received their first diagnosis of tinnitus (ICD-9-CM code 388.3 or ICD-10-CM codes H93.1, H93.11, H93.12, H93.13, or H93.19) at outpatient facilities (doctor's offices or outpatient departments of hospitals) between January 1, 2014 and December 31, 2016. We defined the date of their first-time diagnosis of tinnitus as their index date. We excluded 18,489 patients who had received a diagnosis of major depressive disorder (ICD-9-CM codes 296.20–296.26, 296.30–296.36, 296.82, and 300.4 or ICD-10-CM code F33) or bipolar disorder within 3 years prior to the index date. Ultimately, the selected study cohort included 375,272 patients with tinnitus.

We likewise retrieved patients of a comparison cohort from the registry of beneficiaries included in the NHIRD. After excluding patients aged <20 years of age and those with a history of tinnitus in the prior 3 years, we used propensity-score matching to select 375,272 comparison patients. The matching baseline characteristics were sex, age, monthly income, geographic region, urbanization level of the patient's residence (5 levels, 1 = most urbanized, 5 = least urbanized), selected medical co-morbidities associated with tinnitus (hyperlipidemia, diabetes, coronary heart disease, and hypertension), and index year. The index year for the study cohort was the year when they received their first-time diagnosis of tinnitus. For comparison cohort patients, we defined their index year as the year of their matched tinnitus case after ensuring they had, at least, one episode of medical care utilization in the index year. Furthermore, we assigned their index date as the date of first utilization of medical care in the index year for the comparison cohort. We also ensured that comparison patients had not received a diagnosis of major depressive disorder within 3 years before their index date.

### Outcome Variable

All the patients were tracked for a one-year period, following their index date to identify any claim with a diagnosis of MDD, the outcome variable of interest in this study.

### Statistical Analysis

Statistical analyses were performed using the SAS statistical package (SAS System for Windows, Version 9.4, Cary NC, USA). We carried out Kaplan–Meier analysis with the log-rank test to test differences in 1-year, MDD-free survival between the study cohort and the comparison cohort. Kaplan–Meier estimator is one of the best methods to be used to estimate the survival function from lifetime data. We used Cox proportional hazards regression to estimate the 1-year adjusted hazard of MDD following tinnitus. The Cox proportional hazards model is employed for investigating the association between the survival time of patients and one or more predictor variables. We also verified that the proportional hazards assumption was satisfied based on survival curves of the two strata (study and comparison cohorts) showing proportional separation between the curves over time. We used an adjusted hazard ratio (HR) along with 95% confidence intervals (CI) to estimate the risk of MDD.

Furthermore, despite using propensity-score matching to select the comparison cohort, the large sample sizes used may produce significant *p*-values despite negligible differences in magnitude or composition. We, therefore, measured effect sizes to quantify these differences by calculating Cohen's d, Cohen's h, or Cohen's φ (20). We used a conventional two-tailed value of *p* < 0.05 to assess statistical significance.

## Results

Table 1 presents the baseline characteristics of the study and comparison cohorts. Total sample mean age was 50.09 ± 15.82 years; 50.27 ± 15.77 and 49.91 ± 14.38 years for the study cohort and the comparison cohort, respectively (*p* < 0.001, Cohen's d = 0.02). There were statistically significant differences in sex composition (men, 46.95 vs. 46.45%, *p* < 0.001, Cohen's h = 0.01), monthly income (≥ NT$25,001, 43.68% vs. 44.40, *p* < 0.001, Cohen's φ < 0.01), geographic region (*p* < 0.001, Cohen's φ = 0.01), and urbanization level of the patients' residence (*p* < 0.001, Cohen's φ = 0.01) between the study cohort and the comparison cohort. There were no significant differences in the rates of hyperlipidemia (22.67 vs. 22.69%, *p* = 0.858, Cohen's h < 0.01), diabetes (12.65 vs. 12.78%, *p* = 0.095, Cohen's h < 0.01), and hypertension (25.94 vs. 25.86%, *p* = 0.425, Cohen's h < 0.01). However, there was a significant difference in the rate of coronary heart disease between the study and comparison cohorts (8.13 vs. 8.32%, *p* = 0.003, Cohen's h < 0.01). All effect sizes were all minuscule (≤ 0.02), and, therefore, the significant differences between the cohorts were negligible.

**Table 1:** Demographic characteristics of patients with tinnitus and comparison patients in Taiwan, 2014-2016 (total patients = 750,544).

**Variable**	**Patients with tinnitus** **(*****n*** **=** **375,272)**		**Comparison patients** **(*****n** **=*** **375,272)**		***P* value**
	**Total No**.	**%**	**Total No**.	**%**	
Male	176,190	46.95	174,323	46.45	<0.001
Age, Mean (SD)	50.27 (15.77)		49.91 (14.38)		<0.001
Hypertension	97,344	25.94	97,041	25.86	0.425
Coronary heart disease	30,496	8.13	31,215	8.32	0.003
Hyperlipidemia	85,091	22.67	85,156	22.69	0.858
Diabetes	47,474	12.65	47,956	12.78	0.095
Monthly income					<0.001
< NT$1~15,841	72,064	19.20	69,829	18.61	
NT$15,841~25,000	139,283	37.12	138,815	36.99	
≥NT$25,001	163,925	43.68	166,628	44.40	
Geographic region					<0.001
Northern	182,624	48.66	186,961	49.82	
Central	90,934	24.23	89,875	23.95	
Southern	94,398	25.15	91,795	24.46	
Eastern	7,316	1.95	6,641	1.77	
Urbanization level					<0.001
1(most urbanized)	105,288	28.06	108,435	28.90	
2	111,324	29.66	112,384	29.95	
3	66,312	17.67	65,256	17.39	
4	48,042	12.80	46,275	12.33	
5(least urbanized)	44,306	11.81	42,922	11.44	

*SD, standard deviation*.

**Figure 1:**
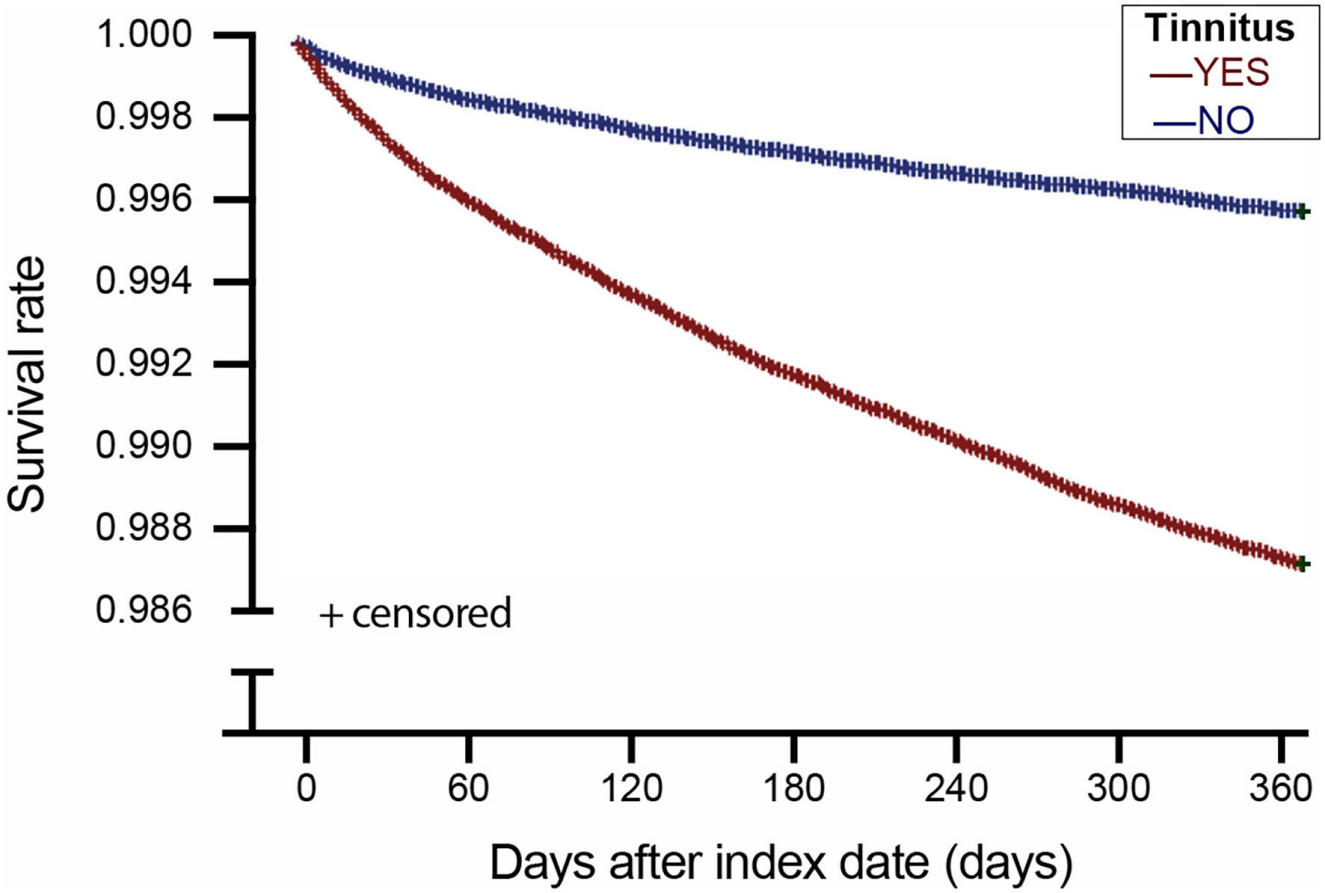
Major depressive disorder-free survival of patients with tinnitus and comparison patients in Taiwan.

Table 2 shows the incidence of MDD within 1 year following the index date. The overall incidence of MDD was.78 (95% CI = 0.76~0.80) per 100 person-years, 1.17 (95% CI = 1.14~1.21) and.38 (95% CI = 0.36~0.40) in the study cohort and comparison cohorts, respectively. Figure 1 displays the results of the Kaplan–Meier survival analysis. The log-rank test was commonly used to compare the survival distributions of two samples. In this study, the log-rank test revealed that the study cohort had a significantly lower 1-year MDD-free survival rate than the comparison cohort (*p* < 0.001).

**Table 2:** Incidence rate, crude, and adjusted hazard ratios for major depressive disorder among study patients.

**Major depressive disorder occurrence over 1-year follow-up**	**Total** ***(n** **=*** **750,544)**		**Patients with tinnitus** **(*****n*** **=** **375,272)**		**Comparison group** **(*****n** **=*** **375,272)**	
	**No**.	**%**	**No**.	**%**	**No**.	**%**
Yes	5,812	0.77	4,382	1.17	1,430	0.38
Incidence per 100 person-years (95%CI)	0.78 (0.76~0.80)		1.17 (1.14~1.21)		0.38 (0.36~0.40)	
Crude HR (95% CI)	-		3.07^***^ (2.89~3.26)		1.00	
Adjusted^a^ HR (95% CI)	-		3.08^***^ (2.90~3.27)		1.00	

*** *Indicates p < 0.001. HR, hazard ratio*.

a *Adjustment for patient's sex, age, urbanization level, monthly income, geographic region, hypertension, hyperlipidemia, coronary heart disease, and diabetes*.

Table 2 further presents the crude and adjusted HRs for MDD during a one-year follow-up. Cox proportional hazard analysis showed that the patients in the study cohort had a higher unadjusted hazard of MDD than the patients in the comparison cohort (HR = 3.07, 95% CI = 2.89~3.26). In addition, the adjusted HR of MDD during one-year follow-up was 3.08 (95% CI = 2.90~3.27) for the patients in the study cohort compared to the comparison cohort after adjusting for age, sex, monthly income, geographic region, urbanization level of the patient's residence, hyperlipidemia, diabetes, coronary heart disease, and hypertension.

## Discussion

To our knowledge, this is the first population-based study, addressing the question of whether prior tinnitus may either trigger or be an initial symptom preceding MDD. We found that the overall incidence of MDD was 1.17 (95% CI = 1.14~1.21) and.38 (95% CI =0.36~0.40) in the study cohort and comparison cohorts, respectively. The adjusted HR of MDD during one-year follow-up was 3.08 (95% CI = 2.90~3.27) for the patients in the study cohort when compared to the comparison cohort. Previous literature has shown an association between tinnitus and depression, but these studies either had small samples or were not population-based samples, impeding generalization of findings to the population level (3). In addition, although a cross-sectional study is not inherently inferior to a cohort study, prior studies were cross-sectional (13, 16, 18, 21, 22), which might not permit the identification of a causal relationship. Furthermore, some studies advocate that tinnitus does not relate to depression (23). The explanation might come from the similarities in the actual diagnosis or measurement of symptoms of both entities (23).

Persons with tinnitus had a 3-fold hazard of occurrence of MDD 1 year compared to those matched to them on demographic and co-morbidity status in this nationally representative Taiwan population sample. Previous studies showed that higher-order psychological/psychopathological aspects may drive effects of tinnitus distress and consequential depression (24, 25). Studies also showed that three facets of personality traits of neuroticism, including anxiety, depression, and emotional volatility, are associated with tinnitus distress over time (8, 26). The elevated MDD risk suggests that physicians should be alert to psychological symptoms in patients with tinnitus, such as depressed mood, decreased interest or pleasure, sleep quality, weight changes, appetite, observable psychomotor slowing or agitation, low energy, ability to concentrate, think or make decisions, thoughts of worthlessness, excessive or inappropriate guilt, recurrent thoughts of death or suicidal ideation, or a suicide attempt. Referral for psychological evaluation may be needed to establish the diagnosis of MDD.

In addition to presenting symptoms, the underlying mechanism between tinnitus and MDD is often convoluted (27). Neuroimaging studies suggest that tinnitus is associated with depressive mood *via* involvement of the nonauditory brain areas, including the anterior parietal area, the limbic system, which consists of the anterior cingulate cortex, anterior insula, amygdala, and the hippocampal and parahippocampal area (14, 28, 29). To begin with, cochlear injury leads to an enhanced “firing rate” in various central auditory pathways. While a tinnitus signal may originate from lesion-induced neural plasticity of the auditory pathways, it can be canceled out at the level of thalamus by an inhibitory feedback connection from limbic regions, which block the tinnitus signal from reaching the auditory cortex (30). Subsequently, altered input causes reshaping of the auditory pathway, leading up to subtle reorganization of non-auditory limbic brain structures and compromising the inhibitory gating mechanism, resulting in chronic tinnitus (30). Decreased gray matter volume has been reported in ventromedial and dorsomedial prefrontal cortices, nucleus accumbens, anterior and posterior cingulate cortices, hippocampus and supramarginal gyrus, amygdala, and the anterior subcallosal angle, which are areas correlated with depression and anxiety (31, 32). Another explanation is the damaging impact of distress caused by tinnitus, activating inflammatory processes by stimulating the formation of proinflammatory cytokines, which may destabilize neurons in the amygdala and prefrontal cortex (33). This may launch pathological provocation of the hypothalamic–pituitary–adrenal axis, altering the chemical balance of the brain to a low noradrenaline and serotonin state in the afferents from the locus coeruleus and raphe nucleus, resulting in the manifestation of depressive symptoms (11).

Our study has several strengths. Taiwan's NHI provides a conveniently accessible and affordable health care system for every resident (with negligible copayments), reducing the potential for diagnostic bias usually associated with socioeconomic status or residential location. Furthermore, because the NHIRD provides coverage for every type of medical care event for all Taiwanese residents with very low copayments, the NHI databases have claims records of ~23 million Taiwanese, including outpatient visits, emergency department visits, and inpatient admissions. Therefore, our study was able to capture data from any source if a diagnosis of tinnitus and MDD occurred. Troublesome tinnitus and MDD are alarming conditions, which usually result in patients seeking immediate medical help, which is facilitated by affordable and accessible health care facilitated by NHI. Therefore, identification of tinnitus and MDD may not vary by socioeconomic status. The use of claims data also avoids possible recall bias associated with self-reported data. Our cohort study design, selecting the comparison group by propensity score matching, strengthens the validity of findings, minimizing selection bias and misclassification bias. In most prior studies, self-reported questionnaires or scales were used to identify depression and tinnitus cases. The validity of disease definition in our study may be more precise.

There are some study limitations. First, the NHIRD claims lack of certain critical items of data, such as lifestyle, dietary habits, body mass index, life events, chronic pain, stress, substance abuse, and family history and genetic factors. In addition, we did not take a sleep problem and medication into consideration. The relationship between tinnitus and MDD reported in this study may be partially explained by the residual confounding of the above factors. Second, some persons affected by tinnitus may not seek medical help because of the impression that treatment of this condition is not effective. The study findings might potentially result in underdiagnosis and undertreatment of tinnitus. However, the possibility of non-differential misclassification in tinnitus could have biased the results toward the null hypothesis. Third, we used medical claims data to investigate the association between tinnitus and MDD in this study. The diagnosis of MDD relied on administrative claims data reported by physicians, which may be less accurate than diagnoses made according to standardized criteria. However, we have assured all the patients with MDD in the present study had at least received one MDD diagnosis from a psychiatrist in which diagnoses were based on DSM-5 diagnostic criteria in Taiwan. Fourth, the NHIRD claims used in this study only included the data interval from years 2011 to 2017. This study was designed as a retrospective cohort study rather than a prospective cohort with a pre-designed protocol, so it should be cautious to draw a causal relationship between tinnitus and MDD. Furthermore, despite being a population-based study, our findings may not apply to other countries because of differences in ethnicity and living environment.

## Conclusions

In this study, we demonstrate that patients with tinnitus are at increased risk of developing MDD. Our findings call for greater awareness of the potential for patients with tinnitus to develop MDD. Careful history taking and timely follow-up to surveil for depressive symptoms are necessary for patients with tinnitus. Health care professionals, such as otolaryngologists, audiologists, and psychologists, should be alert to likely association between tinnitus and MDD and be prepared to address the psychological needs of patients with tinnitus.

## Data Availability Statement

The data analyzed in this study was obtained from the National Health Insurance Research Database, which has been transferred to the Health and Welfare Data Science Center (HWDC). Interested researchers must obtain the data through formal application to the HWDC, Department of Statistics, Ministry of Health and Welfare, Taiwan (https://dep.mohw.gov.tw/DOS/cp-5119-59201-113.html).

## Ethics Statement

The studies involving human participants were reviewed and approved by the Institutional Review Board of Taipei Medical University (TMU-JIRB N202109059). Written informed consent from the patients/ participants or patients/participants legal guardian/next of kin was not required to participate in this study in accordance with the national legislation and the institutional requirements.

## Author Contributions

H-CL, C-HW, and T-HY contributed to conception and design of the study. H-CL organized the database. Y-FC performed the statistical analysis. SX wrote the first draft of the manuscript. H-CL, C-HW, T-HY, and Y-FC wrote sections of the manuscript. All authors contributed to manuscript revision, read, and approved the submitted version.

## Conflict of Interest

The authors declare that the research was conducted in the absence of any commercial or financial relationships that could be construed as a potential conflict of interest.

## Publisher's Note

All claims expressed in this article are solely those of the authors and do not necessarily represent those of their affiliated organizations, or those of the publisher, the editors and the reviewers. Any product that may be evaluated in this article, or claim that may be made by its manufacturer, is not guaranteed or endorsed by the publisher.
